# Evaluation of Serum C-Reactive Protein Levels in Oral Premalignancies and Malignancies: A Comparative Study

**Published:** 2018-11

**Authors:** Sairam Vankadara, Padmaja K, Praveen Kumar Balmuri, Naresh G, Vikas Reddy G

**Affiliations:** 1 Professor and Head, Department of Oral Medicine, G. Pulla Reddy Dental College and Hospital, Kurnool, Andhra Pradesh, India; 2 Postgraduate Student, Department of Oral Medicine & Radiology, G Pullareddy Dental College & Hospital, Kurnool, Andhra Pradesh, India; 3 Professor, Department of Oral Medicine & Radiology, G Pullareddy Dental College & Hospital, Kurnool, Andhra Pradesh, India; 4 Reader, Department of Oral Medicine & Radiology, G Pullareddy Dental College & Hospital, Kurnool, Andhra Pradesh, India; 5 Senior Lecturer, Department of Oral Medicine & Radiology, G Pullareddy Dental College & Hospital, Kurnool, Andhra Pradesh, India

**Keywords:** Leukoplakia, Lichen Planus, Oral Submucous Fibrosis, Biomarkers, Carcinoma, C-Reactive Protein

## Abstract

**Objectives:**

The aim was to evaluate and compare pretreatment serum C-reactive protein (CRP) levels in patients with oral premalignancies and malignancies with that in healthy controls.

**Materials and Methods:**

The study sample consisted of 90 patients of both genders. The subjects were divided into three groups. Group I comprised 30 healthy controls, while group II included 30 patients with potential oral malignancies including leukoplakia, oral submucous fibrosis (OSMF), and oral lichen planus (OLP), and group III included 30 squamous cell carcinoma (SCC) patients confirmed by histopathological examination. All samples were subjected to CRP analysis. Serum CRP levels were quantitatively determined using the automated immunoturbidimetric method.

**Results:**

In group I, CRP levels were ranging from 0.1 to 18.3 mg/l with the mean ± standard deviation (SD) CRP level of 3.88±4.50 mg/l. In group II, CRP levels were ranging from 0.8 to 53.9 mg/l with the mean ± SD CRP level of 5.59±9.86 mg/l. In group III, CRP levels were ranging from 3.3 to 96 mg/l with the mean ± SD CRP level of 31.72±31.01 mg/l.

**Conclusions:**

According to the results, prediagnostic concentrations of CRP are associated with subsequent development of oral cancer and suggest that plasma CRP level is a potential marker of increased risk of cancer.

## INTRODUCTION

Oral cancer is the most common type of cancer in the head and neck region with an annual incidence of 300,000 cases worldwide [1]. This disease is the most common cause of death and morbidity with a 5-year survival rate of less than 50% [1]. Visible changes are detectable in the oral mucosa in the form of white or red patches before the occurrence of oral squamous cell carcinomas (OSCCs) [2]. Prevention and early detection of such potentially malignant disorders (PMDs) have the potential of not only decreasing the incidence but also improving the survival of those who develop oral cancer.

Many researchers have been searching for specific reliable and easily identifiable biomarkers to differentiate cancer patients from healthy individuals and also to detect patients with precancerous lesions who are at high risks of developing cancer [3].

Acute-phase proteins (APPs) are defined as proteins whose concentration is altered by at least 25% in response to inflammation [4]. C-reactive protein (CRP), serum amyloid A (SAA) protein, and fibrinogen are the main APPs [4].

CRP, a typical systemic inflammation marker, was first discovered in the plasma of patients during the acute phase of pneumococcal pneumonia [5]. CRP is produced in hepatocytes in response to inflammatory cytokines such as interleukin (IL)-1, tumor necrosis factor (TNF)-α, and IL-6 [5].

Few studies have demonstrated that elevated CRP levels are associated with an increased risk of malignancy and have been described as a prognostic factor [6]. Raised CRP concentrations have been demonstrated to be an indicator of a poor prognosis for SCC of the esophagus [6]. Nevertheless, few studies are available on oral cancers and premalignant lesions [6]. Accordingly, the present study was conducted to confirm the role of CRP as a reliable, easily identifiable, and less expensive biomarker in the diagnosis of patients with oral premalignancies and malignancies. Also, this study aimed to evaluate and compare pretreatment serum CRP levels in patients with oral premalignancies and malignancies with that in healthy controls.

## MATERIALS AND METHODS

Patients reporting to the outpatient department of Oral Medicine, Diagnosis, and Radiology at G Pulla Reddy Dental College and Hospital, Kurnool, India, were included in this study. Approval from the institutional ethical committee was also obtained (GPRDCH/IEC/2013/008). The study comprised 90 subjects divided into three groups. Group I comprised 30 healthy controls, while group II included 30 patients with PMDs in the oral cavity including leukoplakia, oral submucous fibrosis (OSMF), and oral lichen planus (OLP), and group III included 30 SCC patients.

Patients under treatment for any potentially malignant and malignant diseases, pregnant women, and those having any inflammatory or systemic diseases were excluded from the study.

After explaining the aim of the study, an informed consent was obtained from the patients.

The potentially malignant and malignant diseases were confirmed by histopathological examination. A 2-ml blood sample was collected from each subject using the standard venipuncture technique under aseptic conditions. The collected blood was subjected to centrifugation to separate the serum, and CRP levels were estimated using immunoturbidimetry which is an in-vitro diagnostic assay for quantitative determination of CRP in human serum and plasma [7].

Agglutination occurs when an antigen-antibody reaction takes place between CRP in the sample and polyclonal anti-CRP antibody which has been adsorbed to latex particles. This agglutination was considered an absorbance change with the magnitude of the change being proportional to the quantity of CRP in the sample. The actual concentration was then determined by interpolation from a calibration curve prepared using calibrators of known concentrations. The increase in absorbance at a 572-nm wavelength is proportional to the CRP concentration [8].

The results were tabulated, and statistical analyses were performed using MedCalc software (version 14; Ostend, Belgium). A P-value of <0.05 was considered statistically significant. Comparison of mean values among the groups was made using Kruskal-Wallis test and analysis of variance (ANOVA) with post-hoc Conover test.

## RESULTS

In our study, age distribution in group I was within a range of 20 to 75 years with the mean ± standard deviation (SD) age of 43.63±8.56 years. Age distribution in group II was within a range of 20 to 75 years with the mean ± SD age of 43.67±13.85 years. Age distribution in group III was with a range of 40 to 76 years with the mean ± SD age of 57.17±10.29 years (Table 1).

**Table 1:** Age (years) distribution in the studied groups

	** Min **	** Max **	** Mean **	** SD **
Group I	20	75	43.63	8.56
Group II	20	75	43.67	13.85
Group III	40	76	57.17	10.29

SD=Standard Deviation

Group I included 15 males and 15 females. Group II included 20 males (66.7%) and 10 females (33.3%). Group III included 10 males (33.3%) and 20 females (66.7%; Table 2).

**Table 2:** Gender distribution in the studied groups

	** Group I **		** Group II **		** Group III **	

	** Number **	** Percentage **	** Number **	** Percentage **	** Number **	** Percentage **
** Female **	15	50.0	10	33.3	20	66.7
** Male **	15	50.0	20	66.7	10	33.3

In group I, CRP levels were ranging from 0.1 to 18.3 mg/l with the mean ± SD CRP level of 3.88±4.50 mg/l. In group II, CRP levels were ranging from 0.8 to 53.9 mg/l with the mean ± SD CRP level of 5.59±9.86 mg/l. In group III, CRP levels were ranging from 3.3 to 96 mg/l with the mean ± SD CRP level of 31.72±31.01 mg/l (Table 3).

**Table 3:** Comparison of mean C-reactive protein (CRP) levels (mg/l) among the studied groups

	** Min **	** Max **	** Mean **	** SD **
Group I	0.1	18.3	3.88	4.50
Group II	0.8	53.9	5.59	9.86
Group III	3.3	96	31.72	31.01

P-value	<0.001; Significant			

Post-hoc test result	3>1,2			

SD=Standard Deviation

I: Control Healthy, II: PMDs, III: SCC

Group II included 15 patients with oral leukoplakia (group II A) with the mean ± SD CRP level of 3.35±2.41 mg/l, 10 patients with OLP (group II B) with the mean ± SD CRP level of 10.52±16.19 mg/l, and 5 patients with OSMF (group II C) with the mean ± SD CRP level of 2.44±1.93 mg/l (Table 4).

**Table 4:** Comparison of mean C-reactive protein (CRP) levels (mg/l) among subgroups II A, II B, and II C

	** Min **	** Max **	** Mean **	** SD **
II A (Leukoplakia)	0.8	9.2	3.35	2.41
II B (OLP)	1.3	53.9	10.52	16.19
II C (OSMF)	0.3	5.5	2.44	1.93

SD=Standard Deviation, OLP=Oral Lichen Planus, OSMF=Oral Submucous Fibrosis

## DISCUSSION

In the present study, the age of the patients affected by PMDs ranged from 20 to 75 years with a mean age of 43.67 years. This was in accordance with the findings by George et al [9] who stated that PMDs most commonly affect 50–69-year olds, occurring about five years earlier than oral cancer. This may be due to various etiological factors such as tobacco, alcohol, infection, genetics, immunosuppression, and malnutrition [9]. However, four patients (13%) in group II were below the age of 30 years. Similar findings were reported in the mentioned study, which explained this based on the fact that various extrinsic and intrinsic etiological factors are now more prevalent among younger population [9].

In our study, most of the patients in group III belonged to the older age group, ranging from 40 to 76 years in age (mean age=57.17 years).

According to Radhakrishnan et al [10], oral malignancies occur predominantly in adults, generally in the 5th and 6th decades of life, due to the higher prevalence of risk factors such as tobacco and alcohol.

In our study, group II included 20 male patients (66.7%) and 10 female patients (33.3%). According to George et al [9], PMDs are predominantly seen in males due to the increased habitual use of tobacco and alcohol. Group III included 10 male patients (33.3%) and 20 female patients (66.7%). A similar finding was reported by Tariq et al [11] while conducting a study on CRP levels in patients with malignancies; they reported that 19 patients out of 31 patients were females with a male-to-female ratio of 1:1.6 [11]. According to Watanabe et al [12], oral cancer is generally more prevalent in males, but in high-risk regions like Southern Asia, the rate of occurrence in females is equivalent to that in males. Few authors reported a poor prognosis and lower survival rates in females, attributed to delay in seeking medical care and lower acceptance of treatment [12].

Regarding the clinical types in group II, most patients had leukoplakia (15 leukoplakia patients, 10 OLP patients, and 5 OSMF patients). According to Abidullah et al [13], leukoplakia is the most common PMD affecting the oral mucosa in India, with a prevalence rate of 0.2–4.9%, due to the use of tobacco products in various forms [13]. These lesions are important because of the risk of developing SCC as between 3% and 17.5% of leukoplakias are thought to undergo malignant transformation [9]. In our study, group III included patients with malignancy. All the patients were diagnosed as having SCC on histopathological examination. According to Viviano et al [14], SCC is the most frequent malignant tumor of the oral cavity. The incidence of oral carcinoma is closely correlated with certain customs and habits of different populations, such as smoking and alcohol consumption.

The highest percentages of subjects with oral carcinoma have been recorded in the Indian subcontinent followed by Europe, South America, and Oceania [14].

In our study, most of the patients in group II were having the habit of using tobacco products, either in smoking form or chewable form. This was considered as the main etiology for the development of PMDs. The habits of smoking and alcohol intake were more prevalent compared to chewing tobacco in this group. This can be attributed to the presence of more males in this group. Females prefer tobacco in chewable form to smoking. According to Parlatescu et al [15], smoking is a factor frequently associated with leukoplakia, and this lesion is much more common among smokers than in nonsmokers.

In group III, the majority of the patients were tobacco users in both chewable and smoking forms. Most of them were using a combination of tobacco and quid.

This can be attributed to the presence of more females in group III. One patient developed carcinoma of the lateral border of the tongue due to chronic irritation from a sharp tooth. According to Napier and Speight [16], those who used tobacco in smoking form, chewable form, or both, develop more lesions with an annual incidence rate ranging from 0.52% to 3.02%, depending on the pattern of use. Non-users of tobacco develop fewer lesions ranging from 0.06% to 0.58% [16].

In our study, the most common site of occurrence of PMDs (group II) was the buccal mucosa followed by the tongue and gingivae. According to Neville and Day [17], PMDs were most commonly seen in the buccal mucosa, alveolar mucosa, and lower lip. The above areas were more commonly affected because they were continuously in contact with the etiological agents such as tobacco [17].

In the present study, the most common site of occurrence of malignancy (group III) was the buccal mucosa followed by the tongue, alveolus, and hard palate. According to Viviano et al [14], a common site of OSCC is the tongue followed by the retromolar triangle, buccal mucosa, floor of the mouth, hard palate, and gingivae. According to Radhakrishnan et al [10], the site of origin of oral cancer usually corresponds with the site of placement of betel quid. Patients who simultaneously chewed and smoked tobacco had a tenfold higher risk of cancer of the oral cavity compared to non-chewers and non-smokers. Patients who only chewed tobacco had a six-fold higher risk of cancer, and the patients who only smoked had a threefold increase in the same risk. It has also been demonstrated that the relative risk of chewing betel quid without tobacco for oral cancer is lower compared to chewing betel quid with tobacco. Areca nut and lime exhibited a definite carcinogenic effect even when they were chewed without tobacco [14].

CRP levels have been used to predict the risk of malignancy and to detect its recurrence and prognosis [18]. The levels of CRP alter on a daily basis and increase with age, high blood pressure, smoking, smokeless tobacco use, and alcohol consumption [19].

In the present study, serum CRP levels were elevated in patients with symptoms such as pain and burning sensation in groups II and III. Oliveira et al [20] conducted a study to determine the relationship between inflammatory markers and perceived pain in head and neck cancer (HNC) patients prior to anticancer therapy. Patients experiencing pain had significantly higher levels of CRP (P<0.01) and TNF-α (P<0.05) compared to the controls and asymptomatic patients, thus suggesting significant positive associations between pain and CRP levels [20].

In the present study, serum CRP levels were evaluated and compared among three groups. The mean ± SD CRP levels were 3.88±4.50 mg/l in group I and 5.59±9.86 mg/l in group II. The mean CRP levels were elevated in patients with PMDs (group II) when compared to group I. In group II, patients with OLP showed a higher mean CRP (10.52 mg/l) followed by patients with leukoplakia (3.35 mg/l) and OSMF (2.44 mg/l). The findings were in accordance with the results of a study conducted by Kumar and Bhateja [4] who showed that prediagnostic concentrations of CRP in precancerous patients are strongly associated with subsequent development of oral cancer.

In the present study, CRP values in group III were ranging from 2.6 to 96 mg/l with the mean ± SD level of 31.72±31.01 mg/l. A similar study conducted by Tariq et al [11] demonstrated that higher levels of CRP corresponded with higher TNM staging and poor overall 5-year survival. The study showed that OSCC patients with elevated preoperative serum CRP levels showed the worst prognosis, and almost all of them died within five years, while the patients with normal preoperative CRP were alive even after 5 years of surgical resection [11]. Thus, it can be concluded that patients with elevated preoperative serum CRP levels show poor survival rates compared to patients with normal preoperative serum CRP levels, and elevated preoperative CRP levels are prognostic indicators in patients with OSCC [11].

In our study, CRP concentrations were found to be significantly higher in patients with OSCC (group III) than in healthy controls (group I). The mean CRP level was 3.88±4.50 mg/l in group I and 31.72±31.01 mg/l in group III; the difference between the two groups was statistically significant (P<0.001). This was in accordance with the study conducted by Acharya et al [21], which revealed that CRP was significantly higher in OSCCs than in healthy controls. They observed raised CRP in 70% of OSCCs. The authors also concluded that CRP in OSCCs was associated with clinical nodal status and lymph node metastasis [21].

In our study, CRP concentrations were found to be significantly higher in patients with OSCC (group III) than in patients with PMDs (group II). The mean CRP level was 5.59±9.86 mg/l in group II and 31.72±31.01 mg/l in group III; the difference between the two groups was statistically significant (P<0.001). These results were similar to the findings of a study conducted by Metgud and Bajaj [22] who revealed that mean CRP levels were higher in patients with premalignant oral lesions compared to the controls. CRP levels in OSCC patients were elevated and were associated with advanced tumor stages [22].

In the present study, based on the clinical diagnosis in group II patients, elevated serum CRP levels were evident in speckled leukoplakia and erosive OLP. Similar findings were reported by Kumar and Bhateja [4] who found that mean CRP levels were higher in severe-grade dysplasia, which is indicative of an impending malignancy.

## CONCLUSION

Chronic inflammation plays an important role in cancer initiation, progression, and metastasis. CRP is a nonspecific marker of systemic diseases and inflammation. The present study revealed elevated serum CRP levels in patients with malignancy compared to patients with PMDs and the control group. Also, CRP levels were higher in patients with PMDs compared to the control group, suggesting that CRP is a biomarker that can be used for the assessment of the severity of the disease. Elevated serum CRP levels are associated with poor prognosis; therefore, CRP can contribute toward the management of these diseases. However, it is still unclear whether CRP levels are elevated before the biological onset of cancer or if an elevated CRP level is a risk factor for the development of cancer. Hence, further studies are required to evaluate pre- and post-treatment serum CRP levels in larger sample sizes to determine the disease status.
